# HAPNEST: efficient, large-scale generation and evaluation of synthetic datasets for genotypes and phenotypes

**DOI:** 10.1093/bioinformatics/btad535

**Published:** 2023-08-30

**Authors:** Sophie Wharrie, Zhiyu Yang, Vishnu Raj, Remo Monti, Rahul Gupta, Ying Wang, Alicia Martin, Luke J O’Connor, Samuel Kaski, Pekka Marttinen, Pier Francesco Palamara, Christoph Lippert, Andrea Ganna

**Affiliations:** Department of Computer Science, Aalto University, Espoo 02150, Finland; Institute for Molecular Medicine Finland, FIMM, HiLIFE, University of Helsinki, Helsinki 00014, Finland; Department of Computer Science, Aalto University, Espoo 02150, Finland; Hasso Plattner Institute, University of Potsdam, Digital Engineering Faculty, Potsdam 14469, Germany; Broad Institute of MIT and Harvard, Cambridge, Massachusetts 02142, United States; Broad Institute of MIT and Harvard, Cambridge, Massachusetts 02142, United States; Broad Institute of MIT and Harvard, Cambridge, Massachusetts 02142, United States; Broad Institute of MIT and Harvard, Cambridge, Massachusetts 02142, United States; Department of Computer Science, Aalto University, Espoo 02150, Finland; Department of Computer Science, University of Manchester, Manchester M13 9PL, United Kingdom; Department of Computer Science, Aalto University, Espoo 02150, Finland; Department of Statistics, University of Oxford, Oxford OX1 2JD, United Kingdom; Hasso Plattner Institute, University of Potsdam, Digital Engineering Faculty, Potsdam 14469, Germany; Hasso Plattner Institute for Digital Health at Mount Sinai, Icahn School of Medicine at Mount Sinai, New York, New York 10065, United States; Institute for Molecular Medicine Finland, FIMM, HiLIFE, University of Helsinki, Helsinki 00014, Finland; Broad Institute of MIT and Harvard, Cambridge, Massachusetts 02142, United States

## Abstract

**Motivation:**

Existing methods for simulating synthetic genotype and phenotype datasets have limited scalability, constraining their usability for large-scale analyses. Moreover, a systematic approach for evaluating synthetic data quality and a benchmark synthetic dataset for developing and evaluating methods for polygenic risk scores are lacking.

**Results:**

We present HAPNEST, a novel approach for efficiently generating diverse individual-level genotypic and phenotypic data. In comparison to alternative methods, HAPNEST shows faster computational speed and a lower degree of relatedness with reference panels, while generating datasets that preserve key statistical properties of real data. These desirable synthetic data properties enabled us to generate 6.8 million common variants and nine phenotypes with varying degrees of heritability and polygenicity across 1 million individuals. We demonstrate how HAPNEST can facilitate biobank-scale analyses through the comparison of seven methods to generate polygenic risk scoring across multiple ancestry groups and different genetic architectures.

**Availability and implementation:**

A synthetic dataset of 1 008 000 individuals and nine traits for 6.8 million common variants is available at https://www.ebi.ac.uk/biostudies/studies/S-BSST936. The HAPNEST software for generating synthetic datasets is available as Docker/Singularity containers and open source Julia and C code at https://github.com/intervene-EU-H2020/synthetic_data.

## 1 Introduction

With the emergence of large-scale biobanks, methods to analyse common genetic variants [single-nucleotide polymorphisms (SNPs)] across diverse human populations are in growing demand. This is especially the case for polygenic risk scoring (PRS) methods, which quantify an individual’s genetic risk for a disease or other phenotypic trait (Choi *et al.* 2020). Derived from one’s genotype, well-calibrated PRSs have the potential to be used for risk stratification and prognostic prediction (Choi *et al.* 2020). The utility of PRS has been demonstrated for certain common diseases among European ancestries, on which most genome-wide association studies (GWAS) were carried out (Mills and Rahal 2020), but some studies have highlighted limitations in transferability of PRS across ancestries and different socio-demographic groups (Araújo and Wheeler 2022). Thus, the development of methods that can improve the generalizability of PRSs is needed. At the same time, only a few accessible large-scale biobank datasets exist and most previous PRS methods have been tested and compared in UK Biobank (Pain *et al.* 2021). More diverse biobank datasets are needed, but due to the highly sensitive nature of genetics data, accessing and sharing individual-level data raises privacy concerns. This makes publicly accessible synthetic data a welcome alternative for methods developers.

Broadly, two main approaches have been used to simulate individual level genetic data. Coalescence-based methods, such as Hudson’s ms and msprime (Hudson 2002, Kelleher *et al.* 2016), use demographic models to generate genomes including both rare and common variants. Reference-based approaches use real genomic data (e.g. 1000 genomes or HGDP) to generate synthetic data, but they are not suitable to generate realistic rare variants. There are also methods, such as simGWAS (Fortune and Wallace 2019), that directly simulate GWAS summary statistics. However, many times they do not meet modern demands for methods development based on individual level data. We will focus on reference-based approaches since for PRSs we are mostly interested in common genetic variation, which forms the bulk of complex trait heritability (Yang *et al.* 2010). Moreover, common SNPs, especially Hapmap3 SNPs (International HapMap 3 Consortium 2010), are widely recommended for PRS computation (Wang *et al.* 2023). HAPGEN2 (Su *et al.* 2011) is a widely used tool for genotype and phenotype simulation, which preserves linkage disequilibrium (LD) patterns of real data through a resampling approach based on the Li and Stephens model (Li and Stephens 2003). However, HAPGEN2 lacks computational scalability and flexibility in phenotype generation to simulate certain scenarios of interest for biobank-scale PRS and SNP-based methods development, where the genetic architecture of the phenotype is an essential factor. Recent alternatives include G2P (Tang and Liu 2019) and Sim1000G (Dimitromanolakis *et al.* 2019). Sim1000G is an integrated R package, but is limited to genotype simulation. G2P encompasses both genotype and phenotype simulation, and is highly customizable, but this setup can be challenging for non-expert users. In Table 1, we provide an overview of the characteristics of these different approaches. Without an integrated approach for parameter selection and evaluation of synthetic data quality, it is difficult for end-users to understand the statistical guarantees and reliability of the generated datasets. To the best of our knowledge, there does not exist a software tool implementing an end-to-end pipeline for synthetic data generation, evaluation and optimization.

**Table 1.** Summary of main differences between HAPNEST and alternative synthetic data generation tools.

							Runtime for 100k synthetic samples	
Method	Genotype simulation	Phenotype simulation	Parameter optimization	Evaluation pipeline	Open source	Software platform	∼20k SNPs	∼100k SNPs
HAPNEST	Yes	Yes	Automated	Fidelity (MAF, LD, population structure, nearest-neighbour adversarial accuracy, GWAS results) and generalizability (kinship relatedness)	Yes	Containerized command line tool	15.0 min for one thread, 6.3 min for eight threads	41.6 min for one thread, 11.7 min for eight threads
HAPGEN2	Yes	No	Manual	None	No	Command line tool	36.3 min for one thread, 33.4 min for eight threads	339.2 min for one thread, 280.9 min for eight threads
G2P	Yes	Yes	Manual	GWAS results	No	Java-based GUI	Excluded from comparison	Excluded from comparison
Sim1000G	Yes	No	Manual	None	Yes	R package	Excluded from comparison	Excluded from comparison

To address these limitations, we introduce HAPNEST, a user-friendly tool for generating synthetic datasets for genotypes and phenotypes, evaluating synthetic data quality, and analysing the behaviour of model parameters with respect to the evaluation metrics. HAPNEST simulates genotypes by resampling a set of existing reference genomes, according to a stochastic model that approximates the underlying processes of coalescent, recombination and mutation. Like HAPGEN2, HAPNEST is also based on the Li and Stephens model of LD (Li and Stephens 2003), but HAPNEST additionally models the coalescence age of segments using an approximate model inspired by the sequential Markovian coalescent model (McVean and Cardin 2005). Phenotypes are subsequently assigned to each sample by integrating user-specified genetic, covariate, and environmental effects. Genetic effects are modelled in terms of heritability and polygenicity. HAPNEST enables efficient simulation of diverse biobank-scale datasets, as well as simultaneously generating multiple genetically correlated traits with population specific effects under different pleiotropy models. Moreover, the HAPNEST software includes an extensive workflow for evaluating synthetic data fidelity and generalizability, as well as approximate Bayesian computation (ABC) techniques for analysing the posterior distributions of model parameters to aid model selection.

We compare the performance of HAPNEST with current state-of-the-art genotype and phenotype simulation tools in terms of data quality and computational speed. Furthermore, as a demonstration of the utility of our tool, we show the application of our diverse, biobank-scale synthetic data for evaluating the performance of various PRS methods under different disease models. Our open-source software tool is available at https://github.com/intervene-EU-H2020/synthetic_data, and has also been distributed as Docker and Singularity containers. We have generated 6.8 million common variants and nine phenotypes with varying degrees of heritability and polygenicity across 1 million individuals and made this large synthetic dataset available at https://www.ebi.ac.uk/biostudies/studies/S-BSST936 to encourage standardized evaluation of new statistical methods by the genomic research community.

## 2 Overview of genotype generation methods

**Figure 1.**
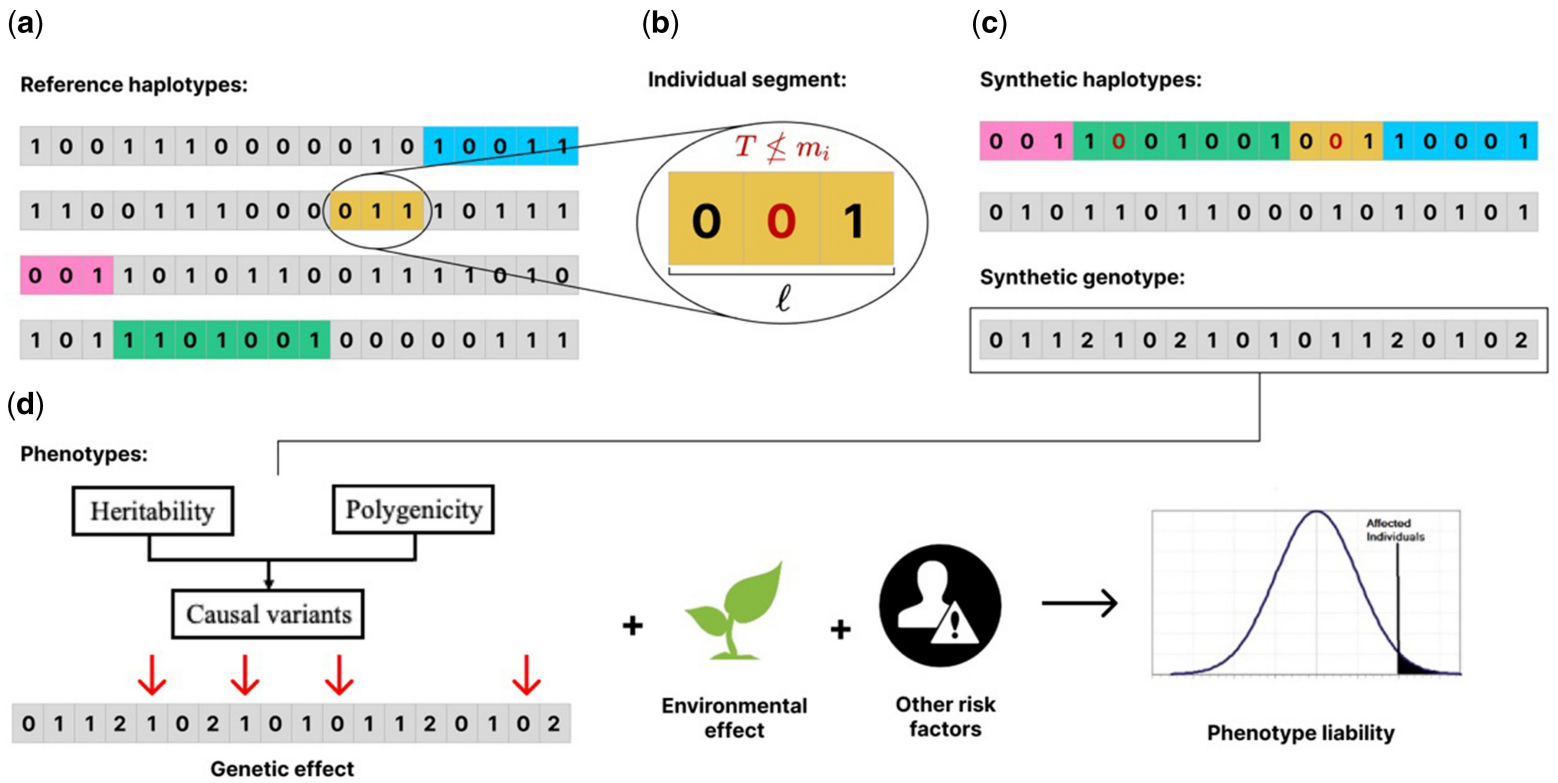
(a) A reference set of real haplotypes, from which segments (coloured) are imperfectly copied to construct a synthetic haplotype. (b) Detailed view of an individual segment. The segment length, ℓ, and coalescence time, *T*, are sampled from a stochastic model. The presence of a genetic variant at position *i* is only copied if $T\leq m_{i}$, where $m_{i}$ is the variant’s age of mutation. Variants that are not copied are shown in red. (c) Synthetic genotypes, *g*, are constructed as pairs of synthetic haplotypes, $h_{j}$, j∈{1,2}. (d) Once the genotype is generated, liability of phenotype will subsequently be assigned to each sample as a summation of genetic effect, covariate effect (if any) and environmental noise.

HAPNEST simulates pairs of synthetic haplotypes, where each haplotype is constructed as a mosaic of segments of various lengths imperfectly copied from a reference set of real haplotypes (Fig. 1a). This resampling approach, based on the Li and Stephens model (Li and Stephens 2003), is also used by other tools such as HAPGEN2, but HAPNEST differs in that it models a varying coalescent age of segments, *T*, when determining genetic distances, ℓ, between cross-over events, and uses this to introduce age-based mutations. This is inspired by the sequential Markovian coalescent model (McVean and Cardin 2005) and motivated by the need to (i) preserve key statistical properties of real genotypes (fidelity) and (ii) limit overfitting to the reference data (generalizability) by introducing variability according to the coalescence process. Previous works have focused on generating high-fidelity data with respect to properties such as LD structure, but the generalizability property is also important when generating a large number of synthetic samples and genetic variants from few reference samples.

Specifically, the real haplotypes to copy from are sampled uniformly from a reference dataset, $D_{s}$, of *k* SNPs for $N_{s}$ samples, limited to individuals belonging to a certain ancestry group *s* (S={AFR, AMR, EAS, EUR, CSA, MID}, where AFR = African, AMR = Admixed American, EAS = East Asian, EUR = European, CSA = Central/South Asian, MID = Middle Eastern). Alternatively, users can specify the proportion of real haplotypes to sample from each ancestry group. We refer to the Discussion section of the paper regarding the complications in interpreting admixed samples. Segments of length ℓ (in centimorgans) are sampled from the real haplotypes (Fig. 1b) based on the model where for group *s*, $\rho_{s}$ is the recombination rate and $N_{e,s}$ is the effective population size. The presence of a genetic variant at position *i* is only copied if $T\leq m_{i}$, where *T* is the segment’s coalescent time and $m_{i}$ is the variant’s age of mutation (obtained from Albers and McVean 2020). This does not create new mutations but introduces variability based on a coalescent model of when mutations entered the population. Two synthetic haplotypes, $h_{j}$, j∈{1,2}, constructed in this way are added element-wise to create a synthetic genotype, *g* (Fig. 1c). For experiments in this text, we consider a reference dataset of 4062 phased genotypes derived from the publicly available 1000 Genomes Project and Human Genome Diversity Project (1KG+HGDP) datasets for six major discrete ancestry groups (Karczewski *et al.* 2020).


(1) $$\ell ∼\;\mathrm{Exp}(2T\rho_{s}),\;T∼\mathrm{Gamma}(2,N_{s}/N_{e,s}),$$

Finally, to aid the scalability of HAPNEST, we develop an efficient multithreaded implementation in the Julia programming language. Specifically, the operations that are parallelized across genotypes include the part of the algorithm that executes Equation (1) to determine how segments should be copied to construct synthetic haplotypes and the I/O operations that write these segments to an output file. We note that this parallelization and use of memory-mapped I/O for constant-time access of reference data makes HAPNEST suitable for use with large reference panels (even if they do not fit in the computer’s memory) and a large number of synthetic samples.

### 2.1 Posterior distributions of model parameters

HAPNEST uses ABC to optimize the model parameters for the fidelity and generalizability objectives (Naeem *et al.* 2020, Alaa *et al.* 2022). We define the ABC summary statistic for the fidelity objective as an LD decay vector, and the summary statistic for the generalizability objective is defined as a vector of the number of duplicate/MZ twin, first-degree and second-degree relatives between the synthetic and reference datasets. We measure generalizability in terms of genetic relatedness (defined by the kinship coefficient), to ensure that the samples in large synthetic datasets are not close copies of samples from the much smaller reference dataset. HAPNEST model parameters are selected as the means of the posterior distributions inferred using emulation-based rejection sampling (Tankhilevich *et al.* 2020). As an illustrative example, Supplementary Fig. S2 shows the posterior distributions of the parameters that best satisfy the multiobjective criteria, for six discrete ancestry groups from the 1KG+HGDP reference for 18 267 HapMap3 variants on chromosome 21. We observe a tradeoff between optimizing the fidelity objective (Supplementary Fig. S3) and optimizing the generalizability objective (Supplementary Fig. S4). This tradeoff can affect the results of downstream analyses such as GWAS (Supplementary Fig. S7 and Table S9), and so it is important for users to choose the objective that matches the priorities of their use case.

## 3 Comparison of synthetic genotype quality

Synthetic data quality is evaluated based on a workflow implemented in the HAPNEST software tool for measuring the fidelity, diversity, and generalizability of synthetic datasets. We compare HAPNEST with three alternative methods: HAPGEN2 (Su *et al.* 2011), G2P (Tang and Liu 2019), and Sim1000G (Dimitromanolakis *et al.* 2019). For these experiments, we consider two models for HAPNEST based on whether the multiobjective (fidelity and generalizability) or LD objective (fidelity only) was used to optimize the parameter values. The evaluation based on the LD objective alone is motivated by the fact that for applications such as GWAS and PRS, it is important to preserve realistic LD patterns in the synthetic data. For the other methods, which do not have built-in optimization procedures, parameters are selected manually to be comparable to the parameters used by the HAPNEST multiobjective approach, where possible (see Supplementary Experiments section for a full list of parameter values used for other methods). The method comparison experiments use $N_{\text{syn}}=1000$ samples generated from a reference of $N_{\text{ref}}=775$ European-ancestry individuals from the 1KG+HGDP datasets (Karczewski *et al.* 2020), for 18 267 HapMap3 variants on chromosome 21. Due to limitations with scalability and compatibility of other methods, we do not evaluate larger synthetic datasets across methods, but we do provide a detailed evaluation of HAPNEST-generated datasets for larger synthetic datasets, non-European ancestry and larger SNP sets: *N*_syn_ = 1000, 25 000, s=EUR,AFR, and *k* = 18 267, 1 09 673 (where the number of SNPs, *k*, correspond to the HapMap3 variants and SNPs with non-zero MAF in all six superpopulations, respectively, on chromosome 21).

### 3.1 Fidelity

Fidelity is measured as the similarity between the real (reference) and synthetic datasets for four properties: minor allele frequency (MAF) distribution, population structure in terms of alignment of the principal components (PCs), LD decay and nearest neighbour adversarial accuracy (full definitions are given in Supplementary Methods sections). The full fidelity results are reported in Supplementary Table S1.

#### 3.1.1 Minor allele frequency

The G2P method had the lowest divergence in MAF between the synthetic and reference datasets, followed by HAPNEST (LD objective), HAPNEST (multiobjective), and Sim1000G (Supplementary Table S1).

#### 3.1.2 LD decay

**Figure 2.**
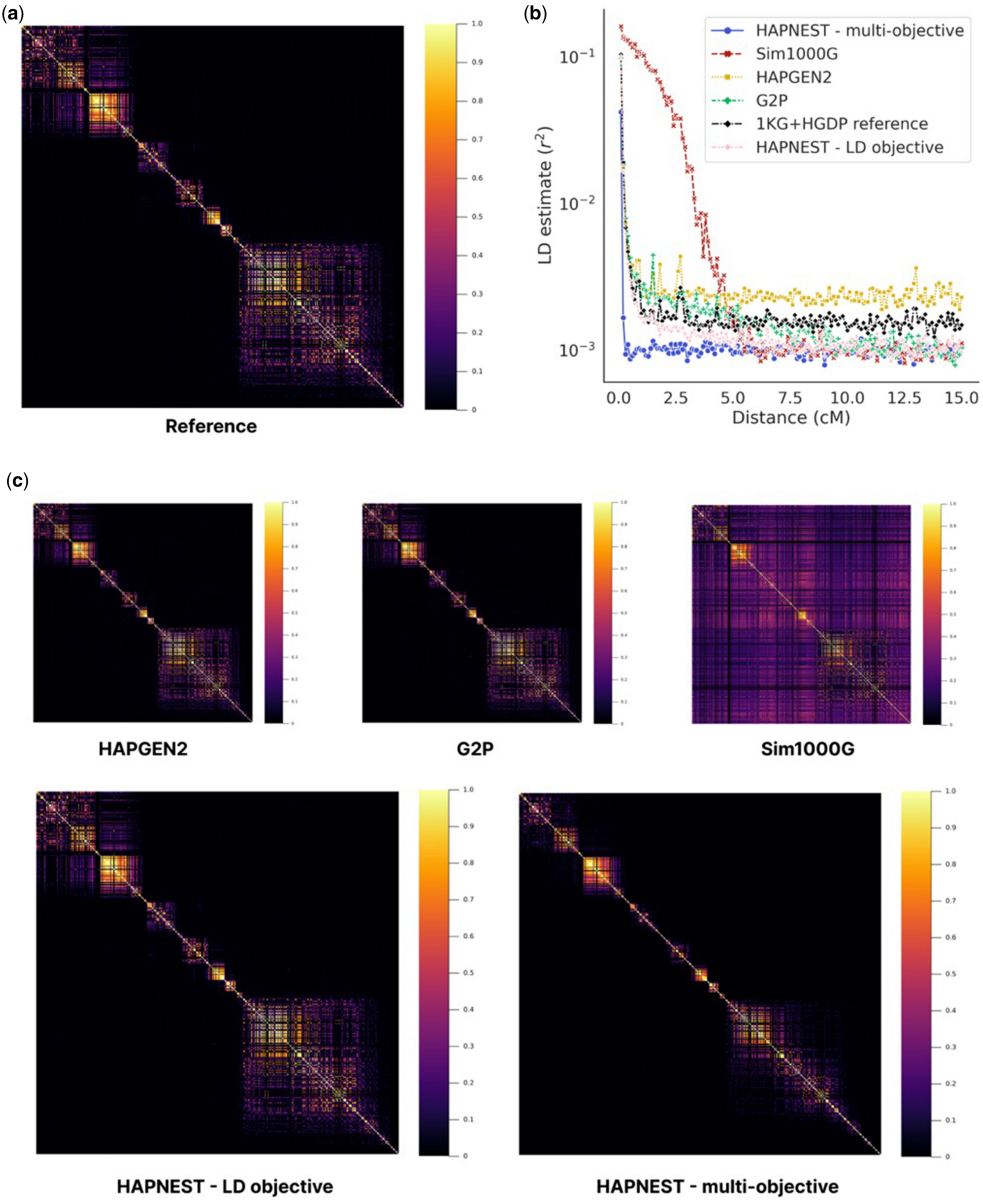
(a) LD correlation for 500 contiguous SNPs selected at random from chromosome 21 HapMap3 variants, for the European-ancestry reference dataset ($N_{\text{ref}}=775$); (b) comparison of LD decay (Laido et al., 2014) for $N_{\text{syn}}=1000$ European-ancestry synthetic samples; (c) comparison of LD correlation (for same 500 SNPs shown in reference panel) for $N_{\text{syn}}=1000$ European-ancestry synthetic samples. We selected alleles with MAF ≥0.001 and used plink with – r2 square flag to compute the LD correlation matrix.

In Fig. 2a and c, we provide a visualization of LD correlation matrices for a snapshot segment of the reference and synthetic samples generated by each method. We observe that the samples produced by HAPGEN, HAPNEST, and G2P faithfully capture the reference LD while Sim1000G amplifies the correlation among SNPs. To quantify this observation, we provide the LD decay plot (Laido *et al.* 2014) for all methods along with the reference in Fig. 2b. The HAPNEST (LD objective) method had the LD decay that least diverged from the reference, followed by the G2P and HAPGEN2 methods (Supplementary Table S1). However, we observe that HAPNEST (multiobjective) has a faster LD decay (Fig. 2b) and more generally, our posterior analysis indicates there is a tradeoff between optimizing the LD and relatedness objectives (Supplementary Figs. S3 and S4). Nevertheless, GWAS results presented later still indicate realistic LD structure at genome-wide significant loci. LD analyses similar to Fig. 2b (Supplementary Fig. S5) and Fig. 2c (Supplementary Fig. S6) for a wider range of HAPNEST-generated synthetic datasets show visually comparable LD results for a larger number of synthetic samples, number of SNPs and non-European ancestry.

#### 3.1.3 Population structure

**Figure 3.**
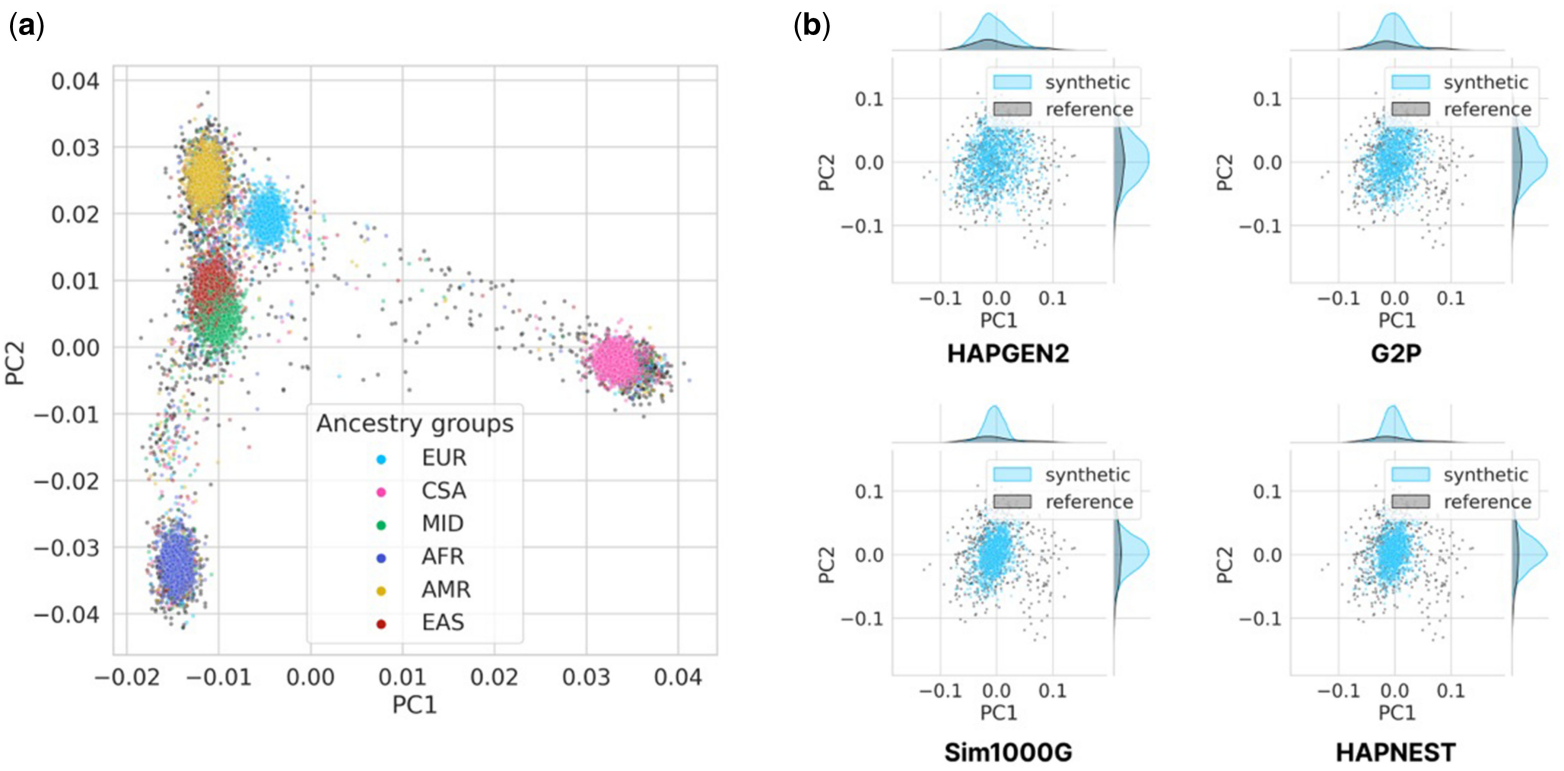
(a) PCA projection plot for *N*_syn_ = 10 002 synthetic samples generated by the HAPNEST method (multiobjective ABC), for chromosome 21 HapMap3 variants, $N_{\text{ref}}=4062$; (b) comparison of PCA projection plots and bivariate densities for $N_{\text{syn}}=1000$ European-ancestry synthetic samples ($N_{\text{ref}}=775$). The highest PC alignment score for preservation of population structure is 0.311 for HAPGEN2, 0.281 (HAPNEST LD objective), 0.222 (G2P), 0.182 (HAPNEST multiobjective), and 0.043 (Sim1000G).

We evaluate preservation of population structure of HAPNEST and other state of the art simulators. Figure 3a shows a PCA projection of 10 002 HAPNEST-generated individuals from six super populations based on chromosome 21. Visual separations between individuals from different populations demonstrate good preservation of overall population structure across multiple ancestries. Figure 3b shows a visual comparison of projecting the top two PCs of 1000 European-ancestry individuals generated by each tool, aligning with the reference. For a more quantitative evaluation, we also compare the PC alignment score, defined as the cosine distance between the first 20 PCs obtained from real and synthetic data within European individuals. The PC alignment score captures the discrepancy in population structure in a high-dimensional space. HAPGEN2 has the highest PC alignment score, followed by HAPNEST (LD objective) (Supplementary Table S1).

#### 3.1.4 Adversarial accuracy

We consider privacy-preserving metrics by calculating the nearest neighbour adversarial accuracy score, which averages the true positive rate and true negative rate for distinguishing real and synthetic data. Adversarial accuracy scores closest to 0.5 are observed for the G2P and HAPNEST (multiobjective) methods, indicating that these synthetic samples are more indistinguishable from the real data (Supplementary Table S1).

Overall, our analysis indicates that no one method performs best across all evaluation metrics, but instead there are tradeoffs that end users should consider, depending on the priorities of their use case. The correlation in the performance of metrics across methods is weak overall, but a lower LD decay distance is generally associated with a higher PC alignment, suggesting that methods with better preservation of LD structure also preserve the population structure of the reference data well (Supplementary Table S1). The nearest neighbour adversarial accuracy is closest to 0.5 for methods that generate high-fidelity data with lower relatedness to the reference data and between synthetic genotypes (Supplementary Tables S2 and S3). We provide further analyses of fidelity and generalizability metrics (Supplementary Table S5 for European ancestry and Table S6 for African ancestry) to demonstrate the properties of HAPNEST datasets for a variety of common use cases. In particular, these results show that for larger synthetic datasets (*N*_syn_ = 25 000, *k* > 1 00 000), HAPNEST achieves comparable performance in the LD metric and better performance in some metrics such as PC alignment, compared with the smaller datasets. Similar results were observed for both the European and African ancestry experiments.

### 3.2 Generalizability and diversity

Diversity is measured by the degree of genetic relatedness (kinship) within the synthetic dataset and generalizability is measured by the degree of genetic relatedness between the real and synthetic datasets. HAPNEST (multiobjective) reached the best generalizability and diversity of all methods evaluated (Supplementary Tables S2 and S3) when considering $N_{\text{syn}}=1000$ synthetic samples. However, it is more appropriate to measure generalizability and diversity on larger and more realistic sample sizes. As there is a limited number of haplotypes in the reference dataset, one might expect that when generating thousands of synthetic samples, some generated genomes might eventually be copies of or highly related with genomes in the reference set. As shown in the next section, scalability is an issue for Sim1000G and G2P, so in this experiment we only consider HAPNEST and HAPGEN2. We quantify the two measurements as for generalizability, where $N_{\text{cross}}$ is the number of closely related pairs (i.e. twins or first-degree relatives, as determined by the kinship coefficient) between the reference and synthetic datasets; and for diversity, where $N_{\text{pairs}}$ is the number of closely related pairs in the synthetic dataset. Supplementary Table S4 shows the generalizability and diversity measurements for HAPNEST in comparison to HAPGEN2, under various sample sizes generated with two reference panels. We observe that HAPNEST also outperforms HAPGEN2 for both generalizability and diversity on larger sample samples.


$$(1-\frac{N_{\text{cross}}}{N_{\text{syn}}*N_{\text{ref}}})\times 100$$

$$(1-\frac{N_{\text{pairs}}}{N_{\text{syn}}^{2}-N_{\text{syn}}})\times 100$$

## 4 Scalability analysis for large sample sizes

The scalability of HAPNEST is validated by measuring the computational speed of generating genotype datasets for a varying number of synthetic samples, SNPs and computing threads. The experiments compare a smaller panel of 18 267 HapMap3 variants and a larger panel of 109 673 variants with non-zero MAF on chromosome 21. The analyses were conducted using a CentOS server with Intel Xeon E5 2680 v3 2.50 GHz processors and 32GB RAM for both single- and multithreaded setups, and use fixed input parameters, $\rho_{s}=2.185,N_{e,s}=500$, to ensure that equivalent setups are being compared for computational experiments. The Sim1000G and G2P methods were excluded from this comparison as they did not scale to the large sample sizes considered here under this setup.

**Figure 4.**
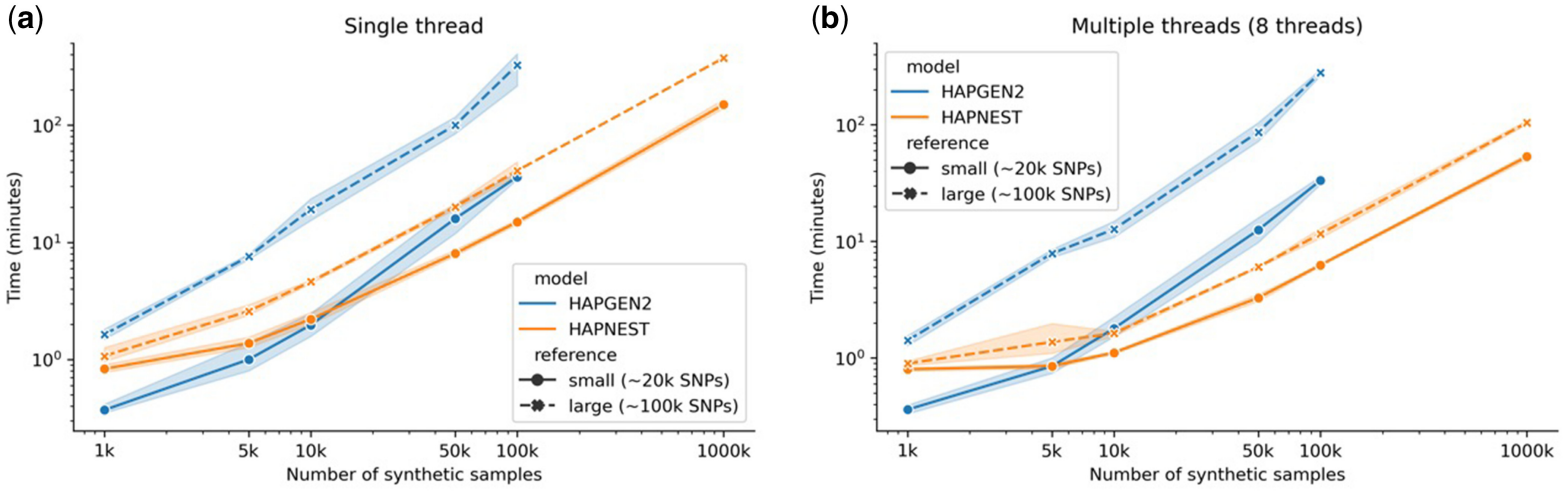
Simulation times for genotype datasets for HAPNEST and HAPGEN2 (other methods are excluded from this comparison due to scalability and compatibility issues), averaged for five trials with 95% confidence intervals plotted, for a varying number of synthetic samples, SNPs and computing threads. Missing results are due to an experiment being terminated for exceeding the memory limit.

We observe that while generation times are similar for smaller datasets, HAPNEST is increasingly faster than HAPGEN2 for more variants and larger sample sizes (Fig. 4) which approach the size of modern biobank-scale genetic datasets. For 100 000 synthetic samples, HAPNEST was between 2.4–8.2*x* times faster than HAPGEN2 (for between 18 267 and 109 673 SNPs) for a single thread and this increased to 5.3–24*x* times faster for multiple threads (tested for eight threads). This demonstrates that HAPNEST uses parallelization to achieve gains in computational efficiency for larger synthetic datasets. We note that HAPGEN2 does not have a command-line option for multiple threads and that similar results for both the single and multithreaded experiments (Fig. 4) indicate that HAPGEN2 was not fully utilizing the available computing resources. The scalability of HAPNEST to generate large datasets under memory constraints can also be attributed to details of our implementation introduced to handle memory-intensive I/O operations, such as batching and memory-mapping. RAM usage statistics reported in Supplementary Tables S10 and S11 demonstrate that HAPNEST utilizes less RAM than HAPGEN2 for larger synthetic sample sizes (3.8–7.9*x* times less RAM for 100 000 synthetic samples on a single thread) and uses multithreading to more efficiently utilize the available computing resources.

## 5 Overview of phenotype generation methods

A continuous or binary phenotype can be assigned to each sample as an aggregation of genetic effect, user-input covariate effect (if any) and environmental noise. The genetic component is generated as a weighted sum of causal allele counts (Fig. 1d). For each causal SNP $\beta_{i}$, the effect size is drawn from a Gaussian distribution with 0 mean and variance determined by three well-studied factors impacting heritability of the variants, the MAF $p_{i}$, local linkage structural $r_{i}$, and the functional annotation $s_{i}$ of the SNP:


$$\beta_{i}∼N(0,{\left[p_{i}(1-p_{i})\right]}^{a}r_{i}^{b}s_{i}^{c}).$$

Power parameters *a*, *b*, and *c* reflect strength of negative selection on each aspect and we used extensive empirical observations (Finucane *et al.* 2015, Gazal *et al.* 2017, Schoech *et al.* 2019) to chose the default parameters. HAPNEST allows SNP’s effect sizes to be drawn from a mixture of distributions with different width, corresponding to variable level of heritability. Our model also allows flexible assignment of individual components’ contribution to the phenotype (heritability), as well as the number of causal variants constituting the genetic risk (polygenicity). We run GWASs for 50 000 synthetic individuals and 1 049 096 HapMap 3 SNPs based on phenotypes generated under different genetic architectures. The Manhattan plots visually resemble Manhattan plots obtained on real data with similar heritability and polygenicity (Supplementary Figs. S8, S9, and S11). Supplementary Fig. S11 shows exemplary GWAS results for traits under two extreme scenarios: low heritability, low polygenicity, and high heritability, high polygenicity. The former resembles phenotypes such as atrial fibrillation and flutter (Supplementary Fig. S8), and the latter resembles typically more heterogeneous traits, such as body pain (Supplementary Fig. S9). Our approach allows us to specify genetic correlations between phenotypes within and, importantly, between ancestry groups.

## 6 Application: comparison of polygenic risk scoring methods

We demonstrated the utility of HAPNEST by comparing seven PRS methods using synthetic data from five ancestry groups. We first generated a synthetic training dataset of 100 000 individuals of European ancestries, and performed a standard GWAS using software plink2 (Purcell *et al.* 2007), correcting for top 20 PCs. We subsequently used the summary statistics to build PRSs in a separate synthetic test set of 25 000 individuals (5000 samples from each ancestry group). To demonstrate variability across genetic architectures, GWAS summary statistics are computed for nine continuous phenotypic traits, with varying heritability (0.03, 0.1, and 0.5) and polygenicity (0.0001, 0.005, and 0.1). We assumed a genetic correlation of 1 across all ancestry groups.

The evaluation of the PRS methods is based on the reference-standardized framework by Pain *et al.* (2021), where for continuous traits, the PRS performance is measured in terms of Pearson correlation between the predicted and observed values. The optimal parameters for each PRS method are identified using cross validation (CV), or pseudovalidation (PseudoVal), if CV is not available.

**Figure 5.**
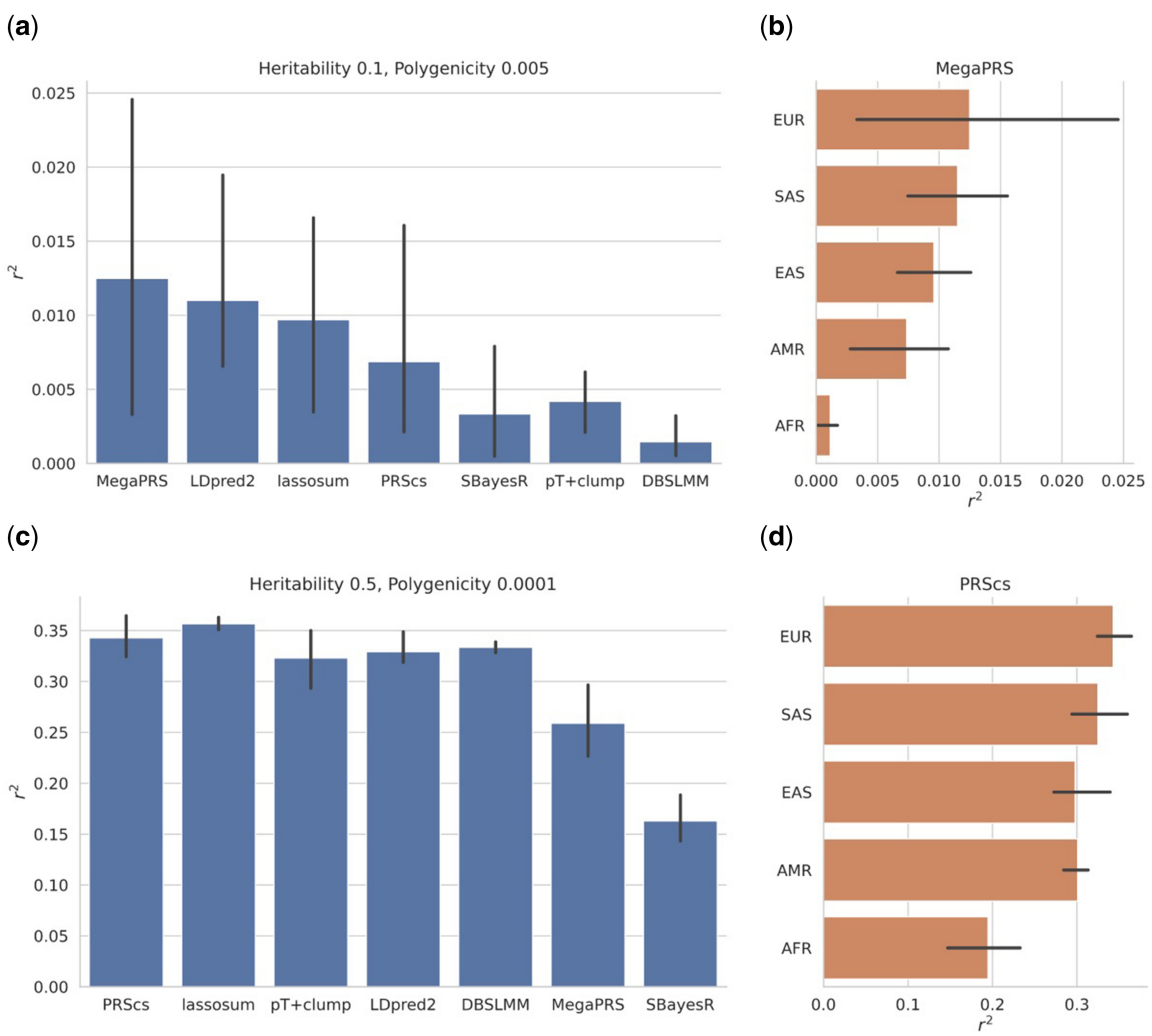
PRS results for two genetic architectures, averaged across three experiment trials with error bars showing the range of outcomes, for HapMap3 variants across 22 chromosomes. (a) Pearson correlation between predicted and observed values, for various PRS methods and a European-ancestry phenotype with heritability 0.1 and polygenicity 0.005. (b) Pearson correlation for various target ancestry groups for the best-performing PRS method (MegaPRS) for the heritability 0.1 and polygenicity 0.005 phenotype. (c) Pearson correlation between predicted and observed values, for various PRS methods and a European-ancestry phenotype with heritability 0.5 and polygenicity 0.0001. (d) Pearson correlation for various target ancestry groups for the best-performing PRS method (PRScs) for the heritability 0.5 and polygenicity 0.0001 phenotype.

Better predictive performance is observed for higher heritability, lower polygenicity architectures (Supplementary Fig. S10). No single PRS method was observed to perform best across all genetic architectures. Methods with sparsity-inducing shrinkage priors (e.g. PRScs) were observed to perform better for higher heritability, lower polygenicity architectures, where genetic effects on most SNPs are zero (Fig. 5c), while other approaches such as MegaPRS performed better for lower heritability, higher polygenicity architectures (Fig. 5a). Multiancestry results replicate known issues with transferability of polygenic risk scores based on European-ancestry summary statistics (Fig. 5b and d).

## 7 Discussion

In this study, we proposed HAPNEST, a new algorithm to generate realistic individual-level genetic and phenotypic data and provide an efficient implementation. HAPNEST meets the demand for diverse, biobank-scale genomic data by improving scalability compared with existing methods. Users can customize population parameters or use parameter estimates derived from the reference dataset. Previous studies have been inconsistent in their approach to evaluating the quality of the generated synthetic data. We provide a comprehensive set of measures to be used for data quality evaluation that have been proposed in the statistical genetics and differential-privacy literature (Yale *et al.* 2019). Genotype generation, phenotype generation and evaluation modules are wrapped in user-friendly Docker or Singularity containers, where each module can be run independently.

Synthetic genotypes are generated by copying and assembling haplotype segments from the reference genome, with distribution of segment length determined by specifics of the target population, including recombination rates, effective population size and samples in the reference panel. Parameters are optimized through the ABC algorithm, which typically results in an output dataset well-balanced across fidelity and generalizability metrics. On top of that, we introduced mutations to the synthetic genome to reduce similarity across individuals. Like HAPGEN2, HAPNEST is also based on the Li and Stephens model of LD (Li and Stephens 2003), but to improve computational scalability and generalizability we have introduced modelling of varying, rather than constant, coalescence time, and the use of mutation ages to determine if mutations are present in synthetic samples.

From our systematic evaluation and experiments, we noticed some general trade-offs in synthetic data quality and in the parameter selection. One trade-off occurs between the preservation of population LD structure and synthetic sample relatedness when constructing large synthetic datasets from much smaller reference datasets. Our observations indicated that parameters optimizing the preservation of LD usually result in higher levels of sample relatedness, as LD typically comes with larger average segment length copied from the reference. On the other hand, shortened segments allow more combinations and higher sample level variability, which results in samples that are less related to each other but increased fragmentation in the LD structure. Furthermore, smaller segments lead to more computational input/output operations when constructing synthetic data files and a slight increase in running time. Segments copied from the reference genome in our algorithm can be conceptually viewed as identity-by-descent (IBD) segments in population genetics (Zhou *et al.* 2020, Browning and Browning 2020). As shown in Equation (1), recombination events ($\rho_{s}$) happen over time (*T*) in the population. Thus, IBD segments degrade over time, which also shows an impact on LD (Thompson 2013, Sticca *et al.* 2021). Our algorithm also provides an implementation of generating “admixed” samples by sampling from multiple reference populations under user defined compositions. However, we would like to note that this approach does not accurately reflect the process of multipopulation diverging and intermixing, therefore it should be used and interpreted carefully.

Compared with other methods, HAPNEST-generated genotypes demonstrated better diversity and generalizability, which are essential features when scaling to large sample sizes. While the genetic relatedness analysis indicated that the genotypes are sufficiently different from the reference data, a nearest-neighbour adversarial accuracy close to 0.5 indicates that statistically speaking, it would be difficult to discern a synthetic genotype from a real genotype. These properties of synthetic datasets are desirable in the context of data privacy, where we may want to create a synthetic twin of sensitive data that preserves key statistical properties of the real data, but cannot be traced back to real individuals. However, we note that we have not tested the HAPNEST method for differential privacy guarantees and for this reason, we advise to use HAPNEST, or any of the reference-based generation methods, only on publicly available genomics datasets.

Once individual level genotypes have been generated, we can subsequently assign phenotypes to each sample as an aggregation of polygenic effects, non-genetic effects and environmental noise. We also implemented population-specific phenotypic effects by assuming shared causal variants across populations with distinct but correlated effect sizes, and multitrait simulation allowing for different genetic correlation and pleiotropy models.

We believe our tool can benefit the community especially for GWAS-related method development, for which one of the examples can be PRS computation and evaluation. HAPNEST allows researchers to assess the validity of genetic scoring methods under a broad variety of setups, including cross-ancestry, trans-diagnostic, and different genetic architectures. Here, as a demonstration of its utility, we applied PRSpipe [PRSpipe is a Snakemake pipeline developed to calculate and evaluate polygenic risk scores from GWAS summary statistics. It implements and extends the GenoPred (Pain *et al.* 2021) pipeline, a reference standardised framework for the prediction of PRS using various state-of-the-art methods] to synthetic data generated by HAPNEST and found that our results, to a great degree, replicated what has been observed by Pain *et al.* (2021). As widely discussed, we found lower cross-ancestry portability of PRSs derived in a single ancestry. For a given phenotype, we set genetic correlations between ancestry groups to 1 and this might be higher than what is observed in real settings and result in slightly inflated trans-ethnic PRS prediction performance. Nevertheless, we still observed reduced prediction accuracy in non-European samples, indicating the synthetic genotype captured the differences of MAF and LD structures across populations. Results under different genetic architectures are concordant with the general expectation: we observe better performance of PRS for phenotypes with higher heritability and lower polygenicity due to the existence of few variants with larger effect that explain large amounts of phenotypic variance. We also noticed that the best performing method can depend on different genetic architecture, reflecting the need for careful considerations when choosing a PRS method. As more studies come online that examine the clinical utility of PRSs, it will be important to have a reference dataset where old and new PRS methods can be compared and their robustness can be assessed as a function of the genetic and phenotypic architecture. We used HAPNEST to create one of the largest genomics synthetic datasets today including 1 million individuals across six major continental ancestry groups, 6.8 million variants, and nine phenotypes. We hope this dataset can generate a reference set for deriving and testing PRS methods within a unified framework.

## Supplementary Material

btad535_Supplementary_Data

## Data Availability

A synthetic dataset of 1 008 000 individuals and nine traits for 6.8 million common variants is available at https://www.ebi.ac.uk/biostudies/studies/S-BSST936. The HAPNEST software for generating synthetic datasets is available as Docker/Singularity containers and open source Julia and C code at https://github.com/intervene-EU-H2020/synthetic_data.
